# Evaluating treatment practices and challenges in systemic Juvenile Idiopathic Arthritis: a comprehensive survey analysis

**DOI:** 10.1007/s10067-024-07111-2

**Published:** 2024-09-28

**Authors:** Xiaohua Tan, Xiaozhen Zhao, Jianghong Deng, Chao Li, Junmei Zhang, Shipeng Li, Caifeng Li

**Affiliations:** Department of Rheumatology, National Centre for Children’s Health, Beijing Children’s Hospital, Capital Medical University, No. 56 Nan Li Shi Lu, Beijing, 100045 China

**Keywords:** Diagnosis, Education, Pediatric rheumatology, Professional development, SJIA, Systemic juvenile Idiopathic arthritis, Treatment

## Abstract

**Objective:**

This study aims to assess current diagnostic and management for systemic Juvenile Idiopathic Arthritis (sJIA) among physicians, evaluate the challenges encountered in diagnosis and treatment, and identify the educational needs and professional development engagements of physicians managing sJIA.

**Methods:**

A nationwide survey was conducted from November 2023 to March 2024 across tertiary and secondary pediatric and general hospitals in China. The survey targeted physicians with at least three years of specialty experience, resulting in 310 valid responses from 25 provinces, autonomous regions, and municipalities. The survey collected data on diagnostic practices, treatment approaches, and professional development related to sJIA. Data collection was facilitated through WeChat, and statistical analysis was performed using descriptive statistics. Ethical approval was obtained from the Ethics Committee of Beijing Children’s Hospital, with informed consent provided electronically by participants.

**Results:**

The survey indicated that all physicians encountered suspected or confirmed cases of sJIA, highlighting its prevalence and the diagnostic challenges associated. Regarding diagnostic standards, 53.9% of physicians used the “Consensus on the Diagnosis and Treatment of sJIA and Macrophage Activation Syndrome,” 18.1% followed the International League of Associations for Rheumatology (ILAR) standards, and 24.8% adhered to the Pediatric Rheumatology International Trials Organization (PRINTO) standards. In treatment strategies, glucocorticoids and IL-6 receptor monoclonal antibodies were extensively used, with the latter receiving “excellent” and “satisfactory” ratings of 46.5% and 36.1%, respectively, demonstrating high efficacy and acceptance. Main challenges included high treatment costs, complexity of diagnosis, patient compliance issues, and potential long-term side effects of biologics. Additionally, 126 doctors (40.7%) actively participated in more than three academic conferences or systematic learning courses related to sJIA, indicating a strong demand for ongoing education, particularly in new treatment developments and diagnostic skills.

**Conclusion:**

The findings emphasize the necessity for standardized diagnosis and customized treatment plans tailored to patient-specific conditions in managing sJIA.

**Table:** 

**Key Points** • *The survey highlights the prevalence and clinical challenges of sJIA among physicians, emphasizing the importance of vigilant diagnosis, multi-system involvement, and differential diagnosis to improve treatment outcomes and patient quality of life.*

## Significance and innovation

### Significance

sJIA is a significant challenge in pediatric rheumatology due to its complex symptoms and severe impact on patients. This study highlights the critical need for improved diagnostic criteria and treatment strategies to enhance patient outcomes. The survey of 310 physicians underscores the prevalence of sJIA and the diagnostic and treatment challenges faced, emphasizing the necessity for ongoing professional education to keep pace with medical advancements.

### Innovation

This study introduces several innovations in sJIA management:

1. Comprehensive survey: Involves a large-scale survey providing detailed insights into current diagnostic and treatment practices.
2. Diagnostic and treatment analysis: Evaluates the use of various diagnostic standards and the effectiveness of treatments, particularly biologics.
3. Professional development focus: Identifies a strong need for continuous education and training among physicians.
4. Clinical challenges: Highlights key challenges such as high treatment costs and patient compliance issues.
5. Data-driven insights: Offer valuable data to inform better diagnostic criteria and treatment protocols.
  Overall, this research provides essential insights and data-driven recommendations to improve the management and outcomes of sJIA, contributing significantly to pediatric rheumatology.

## Introduction

sJIA is a subtype of Juvenile Idiopathic Arthritis (JIA), accounting for approximately 10–20% of all JIA cases, with an incidence rate of about 10 per 100,000. It is characterized by spiking fevers, rash, lymphadenopathy, polyserositis, or hepatosplenomegaly [1, 2]. Managing sJIA requires precise diagnostics and effective therapeutic strategies to mitigate disease progression and improve patient outcomes [3, 4].

Despite medical advancements, sJIA remains a complex disorder with variable clinical presentations, complicating diagnosis and management [3]. The diversity in symptoms often leads to delayed diagnosis and suboptimal care, highlighting the need for better diagnostic criteria and treatment protocols. The introduction of biologics has transformed treatment, offering potential for improved disease control, but their use varies widely among clinicians [5–7].

For certain critically ill sJIA patients, referral for continued treatment is possible. In China, the referral system is supported by mechanisms such as teleconsultation and emergency green channels, which facilitate the timely transfer of patients to appropriate care facilities. These systems are crucial for ensuring that patients receive necessary medical attention efficiently and without delay, thereby improving the overall effectiveness of healthcare delivery.

This study aims to evaluate current diagnostic and treatment practices for sJIA among physicians, identify challenges in management, and assess the educational needs of healthcare providers. By understanding these aspects, the study seeks to pinpoint areas for improvement in training and practice, ultimately aiming to enhance the quality of care for sJIA patients.

## Methods

### Study participants

From November 2023 to March 2024, a survey was conducted across tertiary and secondary pediatric and general hospitals nationwide. Clinical departments included general pediatrics, pediatric rheumatology and immunology, pediatric rheumatology and nephrology, pediatric respiratory and immunology, and adult rheumatology and immunology. The survey targeted physicians with at least three years of specialty experience, including senior residents and above, totaling 323 respondents. A total of 310 valid questionnaires were collected, covering 25 provinces, autonomous regions, and municipalities across the country.

### Survey development

The survey was designed to collect comprehensive information on diagnostic practices, treatment approaches, professional development activities, and educational needs concerning sJIA. It included sections on demographics, professional experience, diagnostic criteria employed, treatment modalities, perceived challenges, and participation in professional development programs. The survey questions combined quantitative and qualitative formats to capture a broad range of relevant data.

### Data collection

Data collection was conducted over a six-month period via WeChat. The survey was distributed through WeChat groups within rheumatology departments of various hospitals, ensuring broad access and easy participation. Participants typically required 5–10 min to complete the survey and submitted it directly through WeChat, with our backend system receiving the responses immediately. Reminders were sent bi-weekly through WeChat to maximize response rates. The survey was designed to be anonymous, collecting no personally identifiable information to maintain confidentiality and encourage honest responses.

### Statistical analysis

Descriptive statistics were used to analyze demographic and professional background data. Frequencies and percentages were calculated for specific diagnostic and treatment practices, as well as participation in educational activities. Challenges in managing sJIA were categorized and analyzed to identify common themes.

### Ethical considerations

The study was approved by the Ethics Committee of Beijing Children’s Hospital. Participants were informed about the study’s purpose and the anonymity of the data collection process before accessing the survey via WeChat. Informed consent was obtained electronically, and participants were assured they could withdraw from the study at any time without consequence.

## Results

### Professional background and qualifications of surveyed medical staff

The majority of doctors surveyed come from tertiary hospitals (287, 92.6%), primarily working in Pediatric Rheumatology and Immunology departments (140, 45.2%). The distribution of titles indicates a significant presence of attending physicians (77, 24.8%) and chief physicians (98, 31.6%), reflecting the high professional qualifications and experience of the doctors participating in the survey.

### Comprehensive overview of sJIA diagnosis, symptomatology, and differential diagnosis in pediatric rheumatology

In the past year, 100% of doctors surveyed reported encountering suspected or confirmed cases of sJIA. Outpatient volume over the past six months ranged from 1 to 1500 cases (10 (4.5, 55)), and inpatient cases ranged from 0 to 1000 (10 (1.5, 35)). Data showed 0 to 300 suspected sJIA cases (5 (2, 11)) and 0 to 160 confirmed cases (3 (1, 10)) over the past year. The proportion of recurrent and treatment-resistant sJIA cases ranged from 0 to 100% (39% (20%, 55%)).

The survey revealed that 78 doctors (25.2%) consider daily intermittent fever as indicative of sJIA, and 191 (16.2%) view relapsing fever as a potential sign. Prolonged fever lasting one to two weeks raised suspicion of sJIA among 258 doctors (83.3%). Rashes were reported as significant markers by 271 doctors (87.4%), and joint pain was noted by 274 doctors (88.4%). Lymphadenopathy and hepatosplenomegaly were considered relevant by 207 (66.8%) and 168 doctors (54.2%), respectively.

In the differential diagnosis of sJIA, 221 doctors (71.3%) prioritized ruling out infections like bacterial, viral, and tuberculosis infections. Malignant tumors, such as leukemia and lymphoma, were the second priority for 121 doctors (39.0%). Autoimmune conditions, including inflammatory bowel disease and systemic lupus erythematosus, were ranked third. Other non-infectious inflammatory diseases, such as CAPS, Takayasu’s arteritis, and Kawasaki disease, were considered by 105 doctors (33.9%).

### Diagnostic practices, challenges, and complication profiles in sJIA

This study gathered data from 310 clinical doctors, revealing varied diagnostic standards for sJIA. Among them, 53.87% utilized the “Consensus on the Diagnosis and Treatment of Systemic Juvenile Idiopathic Arthritis and Macrophage Activation Syndrome (2022 Edition)” [8], 18.06% adhered to the ILAR standards [9], and 24.84% followed the PRINTO standards [10]. Additionally, 3.2% of the doctors were unclear about the specific diagnostic criteria they used.

The time from initial consultation to confirmed diagnosis of sJIA varied, with 41.9% of doctors indicating a diagnosis could be made within 1–2 weeks, 27.1% within 3–4 weeks, and 19.4% within 1–3 months.

Regarding complications, 80.3% of doctors identified macrophage activation syndrome (MAS) as associated with sJIA, with 65.5% estimating its incidence at less than 10%. Other complications mentioned by participants based on their knowledge include joint activity limitations or disabilities (61.9%), liver involvement (52.9%), pleurisy (46.8%), pericarditis (45.2%), uveitis (26.1%), aseptic meningitis (19.7%), thrombocytopenic purpura (16.8%), pulmonary hypertension (15.8%), disseminated intravascular coagulation (15.5%), diffuse alveolar hemorrhage (13.6%), and amyloidosis (2.3%).

The diagnosis process of sJIA, as understood by the participants, presented several challenges. The primary difficulties identified included the need to rule out other diseases, lengthy diagnostic tests, complex clinical manifestations, and challenges in differential diagnosis. Additional factors reported by the participants include a lack of specific biomarkers, insufficient diagnostic experience, cautious decision-making, lack of necessary professional knowledge, deficiencies in hospital diagnostic equipment and methods, and inadequate test precision.

For patients with unclear sJIA diagnoses, the common therapeutic strategies reported by the participants include symptomatic treatment within the department, multidisciplinary consultations within the hospital, online remote consultations, and referrals to superior hospitals. Regarding doctors’ confidence in diagnosing sJIA, 35.8% of participants reported feeling generally confident or fairly confident, 14.5% very confident, and 11.0% not very confident. In assessing the severity of sJIA, 55.5% of doctors reported using the MAS/sJIA scoring system, while 36.1% preferred the Juvenile Arthritis Disease Activity Score (sJADAS).

### Detailed overview of management strategies and clinical insights in sJIA

In the treatment strategies for sJIA, commonly used medications include glucocorticoids, nonsteroidal anti-inflammatory drugs (NSAIDs), disease-modifying antirheumatic drugs (DMARDs), and various biologics, particularly IL-6 receptor monoclonal antibodies and immunosuppressants. Less frequently used drugs include Tumor Necrosis Factor alpha (TNFα) inhibitors, Janus Kinase (JAK) inhibitors, IL-1 receptor antagonists, and thalidomide. The majority of physicians believe that biologic agents are necessary in the treatment regimen.

**Fig. 1:**
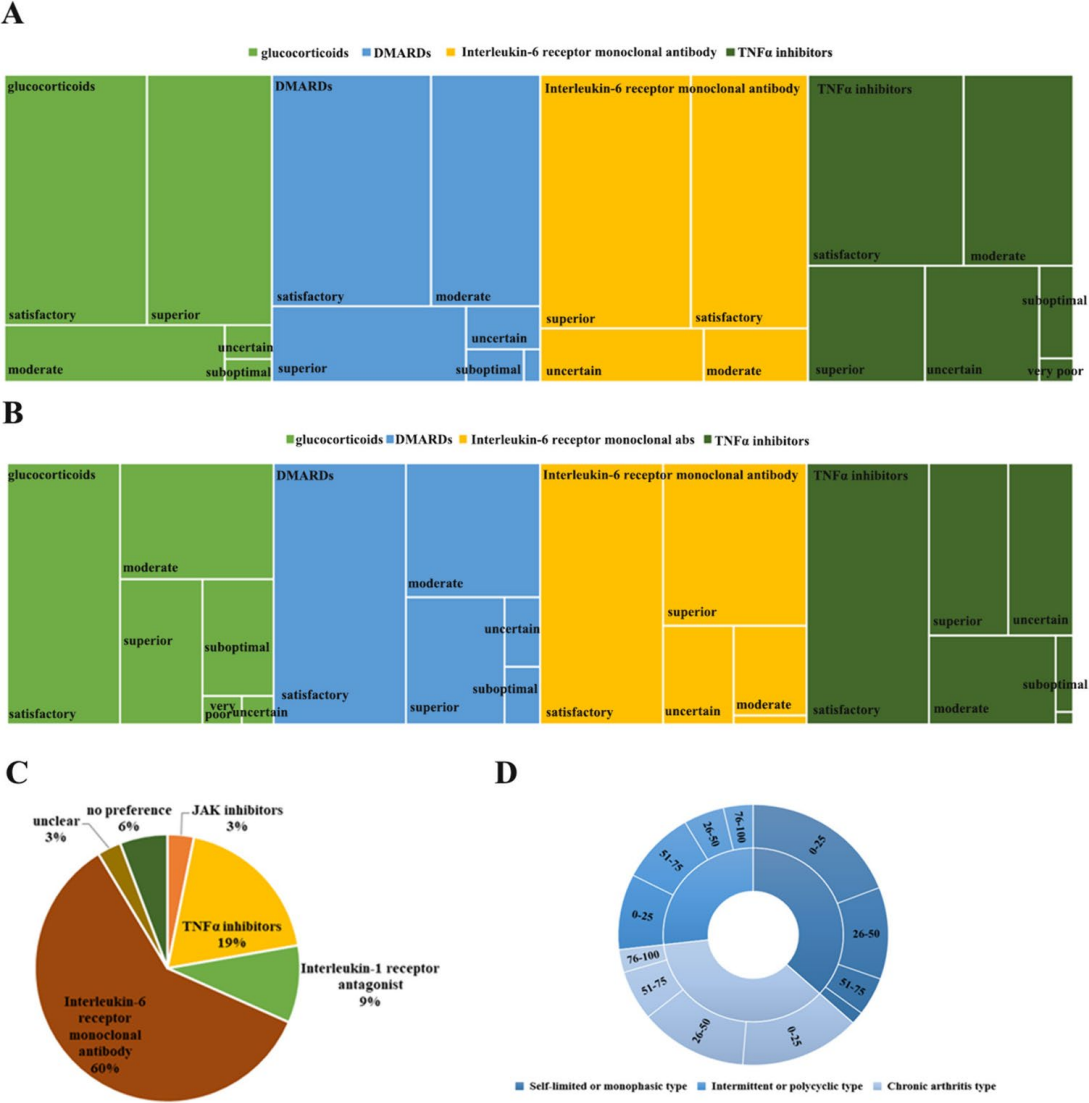
Survey results on treatment practices for sJIA in China.** A** Physicians’ perception of the proportion of sJIA patients treated with biologics: This section displays, through a bar graph, the proportion of sJIA patients treated with biologics as reported by physicians, providing a visual representation of the extent to which biologics are utilized in the treatment of sJIA according to the medical community. **B** Clinical evaluation of efficacy for common sJIA treatment medications: This bar chart assesses the efficacy of commonly used sJIA medications as reported by the surveyed rheumatologists. Ratings range from “very poor” to “superior,” including “uncertain,” “very poor,” “suboptimal,” “moderate,” “satisfactory,” and “superior,” offering a clear visual representation of clinical opinions on treatment efficacy. **C** Clinical evaluation of safety for common sJIA treatment medications: Similar to the efficacy evaluation, this bar chart displays the safety profile of the same medications based on clinical usage. Responses are categorized from “very poor” to “superior,” also including “uncertain,” “very poor,” “suboptimal,” “moderate,” “satisfactory,” and “superior,” highlighting safety considerations in the choice of sJIA treatments. **D** Preferred biologic based on sJIA pathophysiology: This section is illustrated with a pie chart that shows physicians’ preferences for specific biologics considering the pathophysiological mechanisms of sJIA, displaying a distribution from the most to the least preferred biologic agents. **E** Distribution of sJIA onset patterns in China according to clinical experience: This sunburst chart shows the distribution of different sJIA onset patterns as observed by respondents in their clinical practice in China. It provides a detailed quantitative assessment from “monophasic” to “polyphasic,” offering insights into the variability in disease progression

From an efficacy perspective, 43.6% of doctors rate glucocorticoids as “satisfactory” and 38.1% as “superior.” DMARDs receive “satisfactory,” “moderate,” and “superior” ratings from 44.8%, 30.7%, and 17.7% of doctors, respectively. IL-6 receptor monoclonal antibodies receive the highest ratings, with “superior” and “satisfactory” evaluations at 46. 5% and 36.1%, respectively. TNFα inhibitors are generally rated as “moderate,” with 36.1% “satisfactory,” 25.5% “moderate,” and 16.5% “superior” (Fig. 1A).

In terms of safety assessments, the “satisfactory” safety evaluations for lucocorticoids, DMARDs, IL-6 receptor monoclonal antibodies, and TNFα inhibitors are 42.6%, 49.7%, 46.1%, and 45.8%, respectively (Fig. 1B). Among the preferred biologics, 185 physicians (59.68%) consider IL-6 receptor monoclonal antibodies as the first choice (Fig. 0.1C).

Reported unmet clinical needs include recurrent disease difficult to cure completely (71.3%), misdiagnosis or missed diagnosis leading to missed optimal treatment windows (69.7%), high treatment costs (62.6%), the need for long-term glucocorticoid (61.3%), low patient compliance (60.7%), and unclear long-term side effects of biologics (52.9%) (Fig. 1D).

In terms of disease progression patterns, the majority of physicians believe that only 0–25% of sJIA cases are self-limiting or monophasic (161, 51.95%), while the distribution for intermittent or multiphasic types is more even, with 0–25% and 51–75% accounted for by 79 (25.48%) and 76 (24.52%) physicians, respectively. Additionally, 231 doctors (74.52%) consider 0–50% of sJIA cases to be the chronic arthritic type.

### Professional development and educational engagement among physicians in sJIA management

Over the past year, 126 doctors (40.7%) participated in more than three academic conferences or systematic learning courses related to sJIA, either online or offline. Additionally, 121 physicians (39.0%) attended one to two sessions.

In terms of professional education interests, 55 physicians (17.7%) prioritized mastering clinical diagnostic processes, methods, and standards. Guideline interpretation was the top priority for 61 doctors (19.7%), with 50 (16.1%) ranking it second. Standardized treatment and patient management experience were ranked first or third by 56 doctors (18.1%) and 51 physicians (16.5%), respectively.

For research advancements in treatment medications, 52 physicians (16.8%) and 55 (17.8%) considered it the second or third most important topic. Complex clinical case analysis was ranked third in importance by 50 doctors (16.1%). Additionally, 55 physicians (17.7%) rated the basis and standards for clinical medication use as the fourth most important educational content. These findings highlight the specific educational needs of doctors in enhancing their professional knowledge and skills.

## Discussion

This study reveals key challenges and needs in managing sJIA, particularly concerning diagnostic timelines, management of complications, evaluation of treatment medications, and the need for education and professional development.

The findings highlight significant disparities in the time taken from initial symptoms to definitive diagnosis of sJIA. Such variability could be attributed to differences in diagnostic resource availability, the variability in clinicians' recognition capabilities, and unequal distribution of medical facilities by region. Delays can lead to disease progression to more severe stages, raising management complexity and treatment costs. This underscores the necessity to enhance physicians’ capabilities in early detection and intervention through improved education and training.

Of the 10 doctors (3.23%) who were unclear about the sJIA classification criteria, 7 were from general pediatrics, 1 from pediatric rheumatology and nephrology, and 2 from adult rheumatology and immunology. This lack of familiarity may be due to the primary patient populations and focus areas specific to their specialties.

The complexity of managing sJIA is largely dictated by the severity and diversity of its potential complications. Common complications include MAS [11–13], limited joint functionality, and liver involvement [14], with more severe but rare complications such as multi-organ failure also noted. These complications demand that clinicians not only understand sJIA in depth but are also capable of managing these complex clinical scenarios, further emphasizing the importance of continual education in improving patient management quality.

In the treatment of sJIA, the use of biologics such as IL-6 receptor monoclonal antibodies has become pivotal in transforming the therapeutic landscape [5, 15–17]. These treatments are generally highly rated, particularly in controlling disease activity and improving quality of life. However, long-term side effects and high costs remain significant barriers to their broader use. This indicates the need for ongoing evaluation and optimization of treatment protocols, as well as further research into cost-effectiveness and long-term safety.

The survey data indicate a widespread demand among physicians for more education on the latest diagnostic and treatment strategies for sJIA. Physicians seek to update their knowledge through workshops, online courses, and professional conferences, reflecting an ongoing need for professional development. Therefore, developing targeted educational programs and continuing education courses, especially on emerging treatment methods and advanced diagnostic tools, is crucial for enhancing physicians' competencies and ultimately improving patient outcomes.

In summary, effective management of sJIA requires a multifaceted approach, encompassing not just medical treatment but also education, training, and overall patient care. Meticulous consideration and ongoing improvement of these aspects will help enhance treatment efficacy, minimize complications, and optimize patient health outcomes.

## Data Availability

The data are available from the corresponding author upon reasonable request.

## Abbreviations

*sJIA*: Systemic Juvenile Idiopathic Arthritis
*ILAR*: International League of Associations for Rheumatology
*PRINTO*: Pediatric Rheumatology International Trials Organization
*DMARDs*: Disease-modifying antirheumatic drugs
*TNFα*: Tumor necrosis factor alpha
*JAK*: Janus kinase
*IL-6*: Interleukin 6
*MAS*: Macrophage activation syndrome
*sJADAS*: Juvenile Arthritis Disease Activity Score
